# Is Premature Thelarche in the First Two Years of Life Transient?

**DOI:** 10.4274/Jcrpe.709

**Published:** 2012-09-11

**Authors:** Ahmet Uçar, Nurçin Saka, Firdevs Baş, Rüveyde Bundak, Hülya Günöz, Feyza Darendeliler

**Affiliations:** 1 Istanbul University, Istanbul Medical Faculty, Pediatric Endocrine Unit, Fatih, Istanbul, Turkey

**Keywords:** premature thelarche, two years, early puberty, basal LH, growth velocity

## Abstract

**Objective:** Premature thelarche (PT) refers to isolated onset of thelarche in girls younger than 8 years of age. Most cases have an onset under 2 years of age. We aimed to establish whether the onset of thelarche under 2 years of age certifies a transient clinical course, as suggested by several authors.

**Methods:** Sixty-seven girls with an onset of PT under 2 years of age were classified as having early puberty (EP) or classical PT after one year of follow-up. Progression of pubertal findings or absolute growth velocity (GV) standard deviation score (SDS) above 1 SDS constituted the criteria for a diagnosis of EP.

**Results:** Twenty (29.1%) girls were classified as having EP and 47 (70.1%) girls as having classical PT. Basal serum luteinizing hormone (LH; ICMA) values at a cut-off level of 0.3 IU/L were found to be a significant risk factor for having an atypical course [OR=7.8; CI (95%): 2.04– 30.4, p=0.003].

**Conclusions:** Onset of thelarche under 2 years of age does not assure a transient course in a remarkable proportion of girls with PT. An absolute GV value of >1 SDS or a basal LH level ≥0.3 IU/L are suggested as indicators for close follow-up.

**Conflict of interest:**None declared.

## INTRODUCTION

Premature thelarche (PT) is defined as isolated breast development in girls under 8 years of age (1,2,3). Although the etiology of PT is unclear, increased sensitivity of breast tissue to estradiol (E2), transient E2 secretion from ovarian cysts, dietary estrogen intake, and transient activation of the hypothalamo-pituitary-gonadal (HPG) axis have been proposed as possible mechanisms (4,5,6). Girls who receive a tentative diagnosis of PT should be followed up for at least one year to confirm the diagnosis. To date, no laboratory variable has been found to reliably predict which girls with PT will eventually develop early puberty (EP). Hence, careful follow-up has been suggested as the foremost key to diagnosis (7,8,9).

Some girls with PT may show progression of thelarche which may be accompanied by advanced bone age (BA) (3,10). Close follow-up is warranted in such patients, although most remain untreated.

In this retrospective study, we aimed to evaluate whether the onset of thelarche under age two years is predictive of a transient course in the majority of the cases and also to assess the impact of various factors on progression of pubertal findings. 

## METHODS

**Initial Evaluation**

Sixty-seven consecutive girls aged between 5 months and 2 8/12 years who presented to our clinic between May 1998 and September 2010 and who had a presumptive diagnosis of PT at initial examination were evaluated. Birth weight and age at onset of thelarche were extracted from medical records. Being small for gestational age (SGA) and large for gestational age (LGA) were defined as weight for gestation <-2 SDS and >2 SDS, respectively (11). Pubertal staging was done according to the method of Tanner and Marshall by two pediatric endocrinologists (NS and FD) (12). If breast stages differed between the two breasts, the higher stage was taken into account in the evaluation. The same auxologist (MS) evaluated BA readings as well as the weight and height measurements using standard methods. SDS values for weight, height and BMI were calculated using national reference data (13,14). SDS for absolute growth velocity (GV) was estimated using the difference between height measurements at initial evaluation and at the end of the first year of follow-up (15). Target height (TH) was calculated using the formula: maternal height (cm) + paternal height (cm) -13 cm/2. BA and BA-SDS values were evaluated using Greulich and Pyle method (16). In girls with progression of pubertal findings, estimation of predicted height (PH) was done by the Bayley-Pinneau method if the BA was over 6 years (16). The difference between PH and TH was recorded. Results of hormonal assessment including basal serum luteinizing hormone (LH), follicle-stimulating hormone (FSH), E2, as well as the peak LH and FSH responses to gonadotropin-releasing hormone (GnRH) stimulation (sLH and sFSH) were recorded. Serum LH, FSH and E2 levels were measured by immunochemiluminescence assay (ICMA). For LH, the intra- and inter-assay coefficients of variation (CVs) were 4.8% and 10.7%, respectively. For FSH, the intra- and inter-assay CVs were 3.4% and 5.4%, respectively. For E2, the intra- and inter-assay CVs were 4.3% and 7.1%, respectively. Pelvic ultrasonography (USG) evaluations were done by an experienced pediatric radiologist (EY) using a SonoSite Titan ultrasound machine (USA) with a 5 MHz probe. SDSs for mean ovarian volume (MOV) and maximum uterine diameter (MUD) were estimated (17). Ovarian volume and MOV were calculated using the formula for a prolate ellipsoid (length x depth x breadth x 0.523) and the arithmetic mean of the right and left ovaries. Values <2 SDS for MOV and MUD were accepted to indicate prepubertal internal female genitalia (17). The following cut-off values for hormonal and pelvic ultrasound assessment parameters were used to investigate a possible association with clinical findings: basal serum E2 level, 12 pg/mL; serum LH level, 0.3 IU/L; sLH level, 5 IU/L; sLH/FSH ratio, 0.32; MOV SDS and MUD SDS value of +2 SDS (18,19,20).

**Follow-up Data**

The girls with follow-up data of at least one year were considered to have EP if pubertal staging according to Tanner had advanced in the course of the one-year follow-up or if the absolute GV SDS was >1 SDS. Among the girls with EP, those who had a (TH-PH) SDS greater than 1 SDS on further follow-up were considered as potential candidates for compromised adult height.

**Statistical Analysis**

Normality of the variables was tested using the D’Agostino and Pearson omnibus normality test. One-sample t-test and Wilcoxon signed-rank test were used to compare means and medians to a reference value (0), respectively. Student’s t-test and Mann-Whitney U test were used to compare variables in the two groups depending on whether or not the variables were normally distributed. Qualitative data were compared using the chi-square test and Fisher’s exact test. The odds ratio (OR) was used to calculate the risk coefficient of the significant quantitative data. A p-value lower than 0.05 was regarded as statistically significant. The Graphpad Prism 5 statistical package was used for the evaluation.

## RESULTS

**Initial Evaluation**

Data related to history and initial anthropometric measurements are presented in Table 1. Mean age at initial evaluation was 15 (±8.3) months (range 5-28 months). Thelarche had started in the first month of life in 23 (34.8%) girls, between 1 month and 1 year in 33 (48.5%) girls and between 1 year to 2 years in 11 (16.7%) girls. Six (8.9%) girls were older than age 2 years (range 24-28 months) at initial evaluation.

Except for 2 girls born at 32 and 35 weeks of gestation, all subjects were born at term. Birth weight SDS was significantly below the population mean (t=4.3, df=66; p <0.0001). Birth weight was <-2 SDS in 4 (5.9%) girls and >2 SDS in 2 (3%) girls. 

Height and weight SDS values were not significantly different from the population means (p>0.05). BMI was >2 SDS in 6 (8.9%) girls and <-2 SDS in 15 (22.3%). BMI SDS values of the sample were significantly below the population median (p<0.0001).

Initial pubertal staging was B2 in 58 (87.9 %) girls and B3 in 9 (13.4%) girls.

The results of initial laboratory data are also shown in Table 1. BA was >2 SDS in 4 (5.9%) girls. Basal serum FSH levels were significantly higher than LH levels (p<0.0001). Serum E2 levels were >12 pg/mL in 28 (41.7%) girls. Evaluation of pelvic USG findings revealed that MOV was >2 SDS in one girl and that MUD was <2 SDS in all subjects. 

Follow-up 

The girls were followed for a median duration of 34 months (range 13-151 months). At the end of the first year of follow-up, absolute GV SDS was 0.9 ±2.4 (25%-75% IQR; -1.6-1.8). Breast development had progressed to B3 in two girls, regressed in 5 girls and had remained stationary in the remaining girls. According to absolute GV >1 SDS, 20 (29.9%) girls were diagnosed as EP; 2 of these girls also showed advancement of pubertal staging in addition to a GV > 1 SDS. Of the total group, 47 girls (70.1%) were diagnosed as having classical PT. 

There was no significant difference between girls with EP and those with classical PT with respect to being born SGA and age of onset of thelarche (p>0.05). 

As expected, BA SDS values of the girls with EP were significantly higher than those of the girls with classical PT (0.7±1.4 vs. -2±1.4, p=0.0003). Mean GV SDS of the girls with classical PT was -0.2 SDS (range -1.8-0.7) and that of the girls with EP was 2.4 SDS (range 1.1-6.1). 

There were no significant differences between the initial auxological (SDS of weight, height and BMI) and hormonal parameters (basal LH, FSH, E2) nor between pelvic USG findings in the girls with EP and classical PT (p>0.05) (data not shown). GnRH test had been performed in all the girls with EP and in 13 girls with classical PT. Serum sLH and sFSH levels were also not significantly different between the girls with EP and classical PT (5.8±2.1 IU/L vs. 5.7±3.7 IU/L, p>0.05 and 40.8±18.7 IU/L vs. 33.7±21.9 IU/L, p>0.05; respectively). sLH/sFSH ratio was <0.32 in all the girls included in the study, and no differences were detected between the groups (0.15±0.05 vs. 0.17±0.06, p>0.05).

When basal serum LH, E2 and GnRH-stimulated LH levels were compared using the cut-off values described above, a serum LH level ≥ 0.3 IU/L was found to be a significant risk factor for EP [(OR=7.8; CI (95%): 2.04-30.4, p=0.003)]. Comparison with respect to the other cut-off values did not reach significance (p>0.05). 

Pelvic USG findings were also similar between the girls with EP and classical PT (data not shown) (p>0.05).

Girls with classical PT had a median follow-up duration of 37 months (range 13-131 months). There were no significant changes relating to the anthropometric data at initial and final evaluations (data not shown) (p>0.05). In 25 girls with classical PT, breast development regressed in a median period of 13 months (range 6-43 months). Recurrence of breast budding was observed in 7 of these girls after a median follow-up duration of 7 months. Annualised GV values of these girls were < 1 SDS at consecutive evaluations. At the time of our study, menstruation had started in 9 of the girls with classical PT at a median age of 12.1 years (range 11-13.8 years) and median age at onset of menses was similar to that of the general population (12.7±1.1 years) (21).

Mean follow-up duration of the girls with EP was 61 (±31) months (range 13-152 months). The follow-up data of these girls are shown in Table 2. Three of these patients (pts 3, 10, 14) with EP received GnRH agonist therapy due to compromised height potential. Pubarche had started in 6 girls at a median age of 8.4 years (range 7.3 to 9.9 years).

Five of the EP girls (pts 1, 3, 4, 7, 8 and 15) had a history of being born SGA. They all achieved catch-up growth, defined as at least 0.67 SDS increase in weight SDS and height SDS around 3 years of age compared with birth weight and height (22). Two of these girls (pts 3 and 15) were started on GnRH analogue (GnRHa) treatment at 7.3 and 7.7 years of age, respectively.

One patient (pt 10) with an onset of thelarche at 6 months of age was started on GnRHa therapy at 5.1 years of age when pubertal staging was B3 and PH–TH SDS was -1.8 SDS. F

our girls with EP (pts 5,6,12,18) had a cyclic course of breast development although absolute GV SDS values were above 1 SDS during the entire follow-up period, even at the intervals when the breast bud disappeared. Breast development recurred after a median period of 8 months. Another girl (pt 12) had onset of menses at 10.6 years of age when pubertal stage was B2. Her mother’s menarche age was unknown. At 12.5 years of age, this girl was menstruating regularly and her height SDS was normal throughout the follow-up period. 

## DISCUSSION

In this study, we questioned the transient nature of PT in girls with an onset of thelarche under 2 years of age.

We found FSH predominance in all the girls, a finding consistent with previous studies (4,5,6). The use of basal gonadotropin levels (in particular of LH) in the diagnosis has been limited in the past due to poor sensitivity and specificity of the assays. Given these limitations, GnRH-stimulated gonadotropin measurements are commonly used to rule out EP. However, there is a significant overlap of peak LH levels between prepubertal and early pubertal values in normal children (5,23). In studies assessing children with signs of EP, a peak LH value of ≥5 IU/L was found to have a sensitivity of only 78% in predicting subsequent pubertal progression (23). We observed that a basal LH (ICMA) level ≥0.3 IU/L was a significant risk factor for EP. Use of a single basal LH level has significant implications not only in reducing the number of venipunctures and patient discomfort but also in cost. In our study, GnRH-stimulated gonadotropins and the ratio of peak LH/FSH were not significantly different between girls with EP and classical PT at initial evaluation. GnRH testing may be indicated on follow-up when subsequent pubertal development is not concordant with the initial basal LH level. This finding may suggest that basal LH value is a reliable indicator in patients for which closer follow-up is required. A limitation of this study, given its retrospective nature, was that referral for GnRH stimulation testing was made on physician preference, rather than a study protocol. However, the study does reflect the clinical practice from a large tertiary care center. 

Other biochemical markers have been proposed as predictors of pubertal progression. In our study, based on a cut-off point of 12 pg/mL, serum E2 levels at initial evaluation were not significantly higher in girls with EP when compared to those with classical PT (24). This parameter, therefore, does not appear to be a reliable indicator.

At initial evaluation, pelvic USG was also not found to be a reliable indicator of differences between girls with EP and classical PT. In several studies (25,26), pelvic USG parameters were compared with simultaneous hormonal results, and cut-off points for diagnosis of EP were suggested. In a study by Haber et al (25), a uterine volume of 1.8 mL had a sensitivity and specificity of 100%, and an ovarian volume of 1.1 mL had 82% sensitivity and 95% specificity. However, values which appear to be consistent with onset of puberty may be observed in the first year of life, due to the transient physiological activation of the HPG axis. Because some of our patients were infants, we chose to use means and SDs for evaluation, indices which were consistent with prepubertal status in all girls.

Present algorithms assume that onset of thelarche under 2 years of age will have a transient course most of the time (2,27). In our study, we found that 29.9% of the girls with onset of thelarche under 2 years developed EP. Our study is in line with a recent publication by de Vries et al (28), where they evaluated girls with onset of thelarche under 8 years of age and found that the age at onset of thelarche was not predictive of EP. 

The use of anthropometric measurements has been reported to provide more insight as to which children are more likely to develop EP (29,30,31). However, most of the subjects in these studies consisted of girls with onset of thelarche later than 3 years of age. This difference in age distribution may account for the difference in findings related to anthropometric data. Moreover, one of these studies (31) was a cross-sectional study that did not include follow-up of the girls with isolated thelarche. Contrary to their findings, height and weight SDS values of the girls in our study were not significantly different from the population means. The girls in our sample had relatively lower BMI values than the general population mean, which is probably the main issue that poses a diagnostic challenge in this age group. Most of the girls in previous studies had an onset of thelarche after 3 years of age, and central puberty had probably started in a greater proportion of these girls at the time of evaluation. 

BA is an indicator of growth potential, but we believe that it may be more appropriate to use this index in the follow-up of girls after 2 years of age. Because most of the girls in our study were younger than 2 years old, we used GV as a tool for the clinical evaluation and grouping of our patients since GV is regarded as the most sensitive index of systemic estrogen effect (5,10,21). We chose to classify our patients on at least one year of follow-up rather than 6 months since we wanted to define our groups on the basis of absolute GV rather than extrapolation of six-month growth to annualised growth, which would most likely be biased. GV itself is associated with a possible error factor since it is calculated from the difference between two height measurements, thereby combining the imprecision of two readings (32). 

Progressive breast development is associated with EP. However, girls with fluctuating thelarche seem to have two different subtypes with respect to clinical progression: Stanhope (3) identified fluctuating breast development in girls with onset of thelarche under 2 years of age, a finding which was accepted as part of classical thelarche. In another study on fluctuating thelarche (33), the authors reported activating mutations of Gs and suggested a causal relationship with McCune-Albright syndrome (MAS). In our study, the thelarche regressed in 4 girls with EP during follow-up. However, the GV values of the girls were > 1 SDS at the time of breast regression, and breast development reappeared a few months later. In girls with classical PT in our sample who had regression of thelarche during follow-up, annualised GV SDS values were < 1 SDS at all time intervals. We suggest that GV > 1 SDS age be used as a useful predictor for the reappearance of breast budding, and that parents be informed of possible recurrence of the thelarche. 

In our study, the number of girls with a history of being born SGA was too small to consider SGA as a confounding factor (34). The potential influence of environmental factors on early breast development is an ongoing debate. However, discussion of this point is beyond the scope of this presentation. 

**Figure 1:**
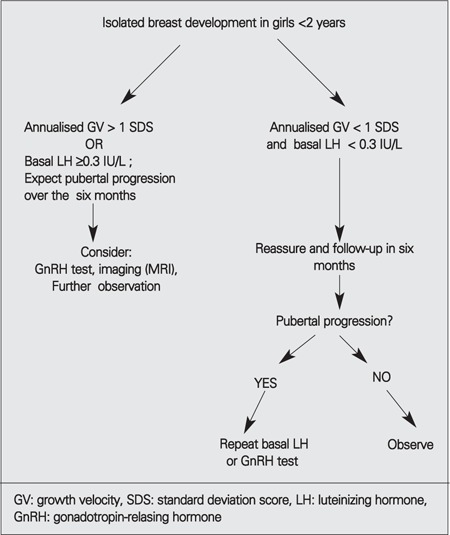
Diagnostic algorithm for girls with premature thelarche under 2 years of age

A limitation of our study is that the length of the follow-up period is relatively short in some of our girls. Based on our findings, we suggest that age at onset of thelarche not be included in the decision-making algorithm and that if absolute GV SDS is >1 and basal LH ≥ 0.3 IU/L, closer follow-up of the girls for progression of pubertal findings is warranted (Figure 1). Because not all the girls who were diagnosed to have classical PT in our study had completed pubertal development at the time of the study, the number of girls with EP could increase. However, such a finding would just increase the proportion of the girls with EP and would reinforce our suggestions for follow-up indications in girls with onset of thelarche in the first 2 years of life. 

In conclusion, we suggest that a significant proportion of girls with onset of thelarche under 2 years of age may develop EP on follow-up. The course can be predicted by assessment of annualised GV and basal serum LH levels.

**Acknowledgements **

We thank Mine Şükür for performing the anthropometric measurements. We also thank Alistair Reeves, Editor in the Life Sciences, for editing this manuscript.

## Figures and Tables

**Table 1:**
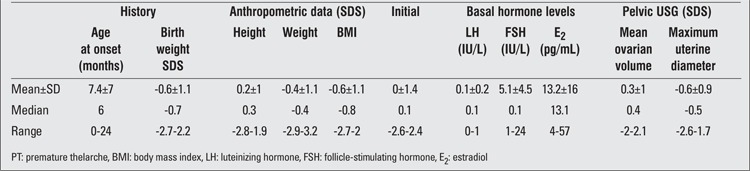
History and initial anthropometric, pubertal and laboratory data of the girls with PT

**Table 2:**
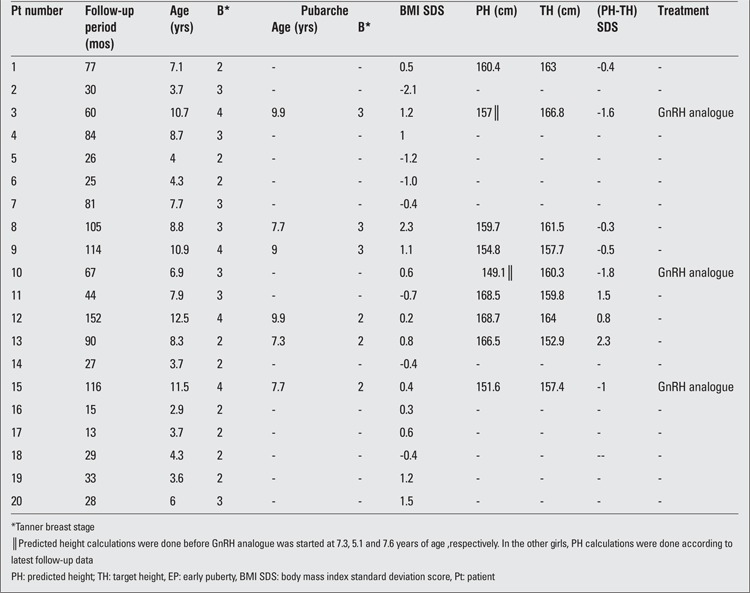
Follow-up data of the girls with EP
